# Oculomotor Behavior of L2 Readers with Typologically Distant L1 Background: The “Big Three” Effects of Word Length, Frequency, and Predictability

**DOI:** 10.3390/jemr18050058

**Published:** 2025-10-18

**Authors:** Marina Norkina, Daria Chernova, Svetlana Alexeeva, Maria Harchevnik

**Affiliations:** 1 Institute for Cognitive Studies, Saint Petersburg State University, 7/9 Universitetskaya Emb., St. Petersburg 199034, Russia; mharchevnik@mail.ru; 2 Center for Cognitive Sciences, Sirius University of Science and Technology, Sirius 354340, Russia; 3 Center for Language and Brain, HSE University, St. Petersburg 198095, Russia; salexeeva@hse.ru

**Keywords:** reading, oculomotor behavior, L2 readers, Russian, word length, word frequency, word predictability, early eye movement measures, late eye movement measures

## Abstract

Oculomotor reading behavior is influenced by both universal factors, like the “big three” of word length, frequency, and contextual predictability, and language-specific factors, such as script and grammar. The aim of this study was to examine the influence of the “big three” factors on L2 reading focusing on a typologically distant L1/L2 pair with dramatic differences in script and grammar. A total of 41 native Chinese-speaking learners of Russian (levels A2-B2) and 40 native Russian speakers read a corpus of 90 Russian sentences for comprehension. Their eye movements were recorded with EyeLink 1000+. We analyzed both early (gaze duration and skipping rate) and late (regression rate and rereading time) eye movement measures. As expected, the “big three” effects influenced oculomotor behavior in both L1 and L2 readers, being more pronounced for L2, but substantial differences were also revealed. Word frequency in L1 reading primarily influenced early processing stages, whereas in L2 reading it remained significant in later stages as well. Predictability had an immediate effect on skipping rates in L1 reading, while L2 readers only exhibited it in late measures. Word length was the only factor that interacted with L2 language exposure which demonstrated adjustment to alphabetic script and polymorphemic word structure. Our findings provide new insights into the processing challenges of L2 readers with typologically distant L1 backgrounds.

## 1. Introduction

### 1.1. Effects of Word Length, Frequency, and Predictability on Reading Behavior

Reading is a complex cognitive process that involves visual word recognition, lexical access, syntactic parsing, and semantic integration, all of which contribute to the goal of text comprehension. The pre-lexical process of word recognition, including visual perception and decoding, is followed by lexical access, which involves selecting a suitable candidate in the mental lexicon [1], while the post-lexical processes of syntactic parsing and semantic integration entail building the sentence structure [2] and integrating different sentence meanings into a coherent understanding of the text [3].

The eye-tracking method is used to investigate cognitive processes underlying reading. Eye movement measures can be divided into two groups according to the processing stages they correspond to: early and late. Early measures include the initial saccade landing position, single and first fixation duration, gaze duration (the sum of all fixation durations on a word during the first pass), the probability of skipping a word, and the probability of fixating a word only once [4]. Late measurements include regression probability, regression path duration (the total duration of all fixations from the first fixation on the word until moving forward), rereading time (the sum of all fixation durations after a regression to the word), and total reading time (the sum of all fixation durations on the word) [4,5]. Early eye movement measures are considered primarily to reflect processing up to the stage of lexical access, while late measures are considered to reflect the processes of syntactic, semantic, and discourse integration [4,5,6].

Decades of research have demonstrated robust effects of word length and word frequency—intrinsic word properties—as well as word predictability—an extrinsic property determined by the sentential context—on eye movement measures [7,8,9]. Together, these properties are termed “the big three”. These effects have been demonstrated across typologically diverse languages, and therefore are supposed to be universal.

Word length primarily influences early reading processes, and it is reflected in early eye movement measures such as first-fixation and gaze durations, as well as the late measure of total viewing time [9,10,11]. In general, longer words tend to result in longer and a greater amount of fixations compared to shorter words [12,13,14]. These findings have been demonstrated in alphabetic languages with diverse orthographic systems, such as English [15,16,17,18,19,20], German [21], French [22], and Russian [23], as well as in non-alphabetic languages, including abjad Hebrew [24], Arabic [25], logographic Japanese [26], and Chinese [27].

The second universal factor that influences word recognition during reading is frequency. Frequent exposure to a word in different contexts determines the rate at which its orthographic, phonological, and semantic representations are activated. Many studies have consistently demonstrated the word frequency effect: when word length is controlled, readers look longer at low-frequency words than at high-frequency words [28,29,30,31]. The frequency effect is robust and has been replicated in both alphabetic and non-alphabetic languages: English [32], Spanish [10], German [9], Dutch [33], Russian [23], Korean [34], and Chinese [35]. Frequency primarily influences earlier language processing, such as lexical access, but also impacts later stages like semantic integration, and it has been examined in various measures [9].

During reading, we anticipate upcoming words. The easier a word is to predict from the preceding context, the faster it is processed [2,36,37]. Predictability has been shown to affect not only early pre-lexical and lexical processing [2,9,38] but also late integration processes [39]. Each new unit of the information must be incorporated into the discourse; when readers accurately predict words, it facilitates smoother and faster semantic integration, allowing them to connect ideas and maintain comprehension. The predictability effect has also been demonstrated for different languages, including French [40], Chinese [38], and Russian [23]. However, data on the timing of predictability effect are controversial [41]. Some studies show that the effect is revealed in later stages of linguistic processing, reflected in late eye movement measures like second-pass reading time, total reading time, and regression rates [10,42]. In contrast, others also report its influence on early measures, such as first fixation duration [43,44,45] or skipping probability [46].

Beyond universal parameters, language-specific factors, such as the type of writing system and grammatical structure, also play a significant role in word processing. Recently, language-specific oculomotor reading patterns were extensively covered in the Multilingual Eye movement Corpus (MECO) study, which initially included 13 languages, with a growing number continually being added [47]. The findings reveal that certain languages stand out, such as Norwegian, Estonian, and Korean. Norwegian readers make shorter and fewer fixations and have a higher skipping rate. Conversely, Estonian language readers make a large number of fixations on the words with relatively long fixations and a high rereading rate. In Korean, where words are short in length (as in Chinese), reading time is short and the skipping rate is high [47]. Furthermore, in the MECO study, the cross-linguistic variability in reading performance was explored. The skipping rate had the strongest systematic variability, with 46% of the variance explained by language differences. Language also accounted for 24% of the variance in the number of fixations during the first run. It accounted for a smaller portion of the variance in duration metrics: first fixation duration (5%), gaze duration (16%), and total fixation time (13%) [47]. This pattern suggests that the majority of cross-linguistic variations in oculomotor behavior at the word level demonstrate the spatial distribution of fixations across words, specifically regarding which words capture attention and which do not. The authors attribute the skipping rate to one of the key predictors of reading: word length. A strong negative correlation was found between a language’s average word length and its estimated skipping rate. For example, Finnish, with a mean word length of 7.82 characters (SD = 3.90), has an estimated skipping rate of 6%, while Korean, with a mean word length of 2.92 characters (SD = 1.27), has a skipping rate of 29% [47].

To sum up, the effects of word length, frequency, and predictability appear to be universal across different languages; at the same time, they are related to language-specific factors. For instance, a language’s average word length is often influenced by its grammatical structure: agglutinative languages tend to have longer words, as they combine multiple morphemes into a single word form, while analytic languages tend to have shorter words, relying on word order and auxiliary words to convey grammatical relationships. Frequency rates highly depend on the degree of inflection in a language (in morphologically rich languages we distinguish between lemma frequencies and wordform frequencies). Predictability, in turn, is influenced by factors such as homonymy rates and word order flexibility (e.g., fixed or free). Another crucial language-specific factor affecting oculomotor reading behavior is the writing system, including the degree of grapheme visual density and orthographic transparency. Therefore, although overall eye movement patterns across languages may appear quite similar and could be claimed as universal, language-specific reading strategies also play a significant role. This underscores the considerable value of cross-linguistic studies.

### 1.2. Reading Behavior in L2

Bilingual research mainly focuses on the mechanism of competition and coordination during the processing of an L1 and an L2. Among the factors that influence reading patterns are individual differences in language proficiency and language usage. L2 reading studies either use a within-subject design, comparing L1 and L2 reading patterns of one and the same samples [33,48] or a between-subject design, comparing the reading patterns of L2 readers with those of L1 readers [49], or a combination of these two approaches [50]. A scoping review by Quiñonez-Beltran and colleagues [51] underlines that L1 characteristics are to be taken into account when planning L2 reading studies design. For instance, a comparative study [52] with Chinese and Arabic readers of English demonstrates that L1 background affects L2 oculomotor reading behavior, namely fixation duration.

It should be noted that most corpora of eye movements, which allow researchers to explore large datasets and a variety of factors affecting oculomotor behavior, include only L1 data, with few exceptions like GECO [21], MECO [50], and CELER [49].

The GECO [21,33] investigates differences between L1 and L2 reading using eye movement measures. The researchers examined unbalanced L1 Dutch–L2 English readers and discovered that these individuals exhibited longer total reading times, increased average fixation durations, a higher number of fixations, and a reduced likelihood of skipping words when reading in their non-dominant language. This oculomotor behavior parallels that of young monolingual children who are just learning to read [53,54] or low-literate monolingual adults [55,56].

The MECO-L2 [50] comprises eye movements during English L2 reading by native speakers of 13 typologically different languages. Participants were tested on component skills such as vocabulary size, spelling, decoding efficiency, and print exposure. The study focuses on the role of L1 in L2 English reading. The contribution of different component skills is further explored in the CELER corpus [57], which includes eye movement data from English L2 readers from diverse linguistic backgrounds (Arabic, Chinese, Japanese, Spanish, and Portuguese), alongside a control group of L1 English readers. CELER also includes scores from standardized L2 proficiency tests. Direct effects of L2 proficiency on oculomotor reading behavior were demonstrated by Berzak, Katz, and Levy [58]. Their findings revealed that first fixation duration and total reading time not only correlated with scores on standardized English proficiency tests but also effectively predicted test outcomes. Eye movements in low-proficient L2 readers were characterized by longer fixation durations, a lower skipping rate, a higher probability of regressive saccades, and more pronounced frequency effects.

It was shown that L2 readers exhibit many qualitative effects similar to those of L1 readers. Notably, however, they demonstrate a so-called proficiency-sensitive lexicon–context tradeoff [21,49]: the eye movements of the most proficient L2 readers resemble those of L1 readers, whereas as L2 proficiency decreases, the readers become less responsive to a word’s predictability based on context and more influenced by word frequency, which remains constant across contexts. This tradeoff reinforces an experience-based explanation for how context-driven expectations are utilized in L2 language processing: diminished exposure and practice result in weaker connections between wordforms and their mental representations. Consequently, accessing vocabulary in the non-dominant language becomes less efficient, as evidenced by greater frequency effects and longer reading times. The less language experience a reader has, the more they rely on context-independent information (e.g., word frequency). As their experience increases, their predictions become more contextualized.

Whitford and Titone [59,60,61], who studied late L2 bilinguals of various ages and different language dominance (English or French), found the same influence of L2 exposure on the strength of the frequency effect in both early and late eye-tracking measures. However, the strength of the predictability effect was not shown to differ in L1 and L2 reading. Mor and Prior [48] reported that the predictability effect was more pronounced in L2 reading while Berzak and colleagues [49] showed that predictability effects were, in L2 reading, larger for gaze duration and total reading time, although evidence for a lexicon–context tradeoff was demonstrated in the following: the word predictability effect on total reading time in L1 was larger than word frequency effect, while in L2 it was vice versa. Fernandez & Allen (2025), comparing the L1 English reading and L2 English reading of German learners, demonstrate a predictability effect on early measures—gaze duration and skipping rates [62]. The effect is found in both L1 and L2 groups, with L2 readers being less efficient. This quantitative, but not qualitative, difference is explained by the reduced capacity for parafoveal processing in L2 readers. The study of Xiao and colleagues with Tibetan–Chinese bilinguals [63] also demonstrates that the preview benefit is more prominent for L1 than for L2 readers and emphasizes that semantic information in L2 reading is accessed at a later stage.

Regarding word length, Cop and colleagues [21] demonstrated that a greater number of symbols in a word leads to an increased fixation count, an effect that is more pronounced even in proficient (B2–C1) L2 readers in comparison with native speakers. Thus, the big three effects in L2 reading depend on the proficiency level; length and frequency effects are more pronounced in L2 reading than in L1 reading, whereas the data on the predictability effect are more controversial.

The vast majority of L2 reading studies focus on reading in English, so eye movement patterns remain understudied cross-linguistically. Daniels and Share [64] emphasize that existing reading theories mostly rely on English reading data and features of English orthography; therefore, taking other writing systems into account can broaden the scope of reading studies and shed some light on universal and language-specific reading mechanisms.

Our study aims to address this gap by presenting data from typologically distinct L1/L2 pairs: native Russian L1 readers and Chinese L2 readers of Russian. These two languages are particularly interesting to compare because they exhibit important differences on two basic levels: writing system and grammar.

### 1.3. Reading in Russian and Reading in Chinese

Eye Movements in Reading Chinese

Modern written Chinese consists of horizontally arrayed strings of characters, going from left to right. Each character occupies a rectangular region of the same size and can be further decomposed into component radicals or even further into a series of individual strokes. Chinese characters typically map onto one-syllable morphemes. Chinese words can be either monomorphemic (one character) or polymorphemic (composed of two or more characters). According to the Chinese Word Corpus of Academia Sinica Taiwan (1998), over 76% of the words (type frequency) consist of two or three characters [65]. When token frequency is considered, words contain one and two characters for 54% and 42%, respectively [61]. Chinese lacks spaces between words, and many cases involve word boundary ambiguity. This makes word segmentation—the extraction of words from a character string—more difficult than in alphabetic languages.

The body of work on eye movement studies on Chinese has been growing in the past two decades. For characters, the effects of character orthography, frequency, and complexity (i.e., the number of strokes) on fixation durations have been obtained [35,66,67,68,69]. For words, the effects of word frequency and word space availability have been demonstrated [38,70,71,72,73,74]. For sentential constraints, the interaction of word ambiguity and context in the lexical ambiguity resolution of Chinese homographs was shown in [75]. These results demonstrate that Chinese readers utilize both word-level and contextual information during reading.

Pan and colleagues [27] developed the Beijing Sentence Corpus (BSC) and presented the eye-tracking data from Chinese readers, including large-scale predictability norms and their effect on saccade targeting. The results showed that low frequency, low contextual predictability, and high visual complexity may lead to difficulties in parafoveal word segmentation, resulting in fixation locations shifting from the center of the word toward its beginning.

Chinese is visually dense in contrast to alphabetical languages, which are typically more horizontally expansive. This difference affects eye movement patterns [76]: Chinese readers make fewer but longer fixations that are positioned closely together, whereas Finnish readers make more numerous but shorter fixations spaced further apart [76]. Also, average fixation durations are longer in Chinese compared with English [76]. Other findings indicate that rightward saccade length is also affected by language. Chinese readers make the shortest forward saccades, while English readers make the longest—even longer than Finnish readers [77]. The average forward saccade length for Chinese readers was half that of readers of the two alphabetic languages [77]. Chinese readers also make more regressions. One of the reasons may be because this is a compensation for frequent word skipping during first-pass reading (more than half of the words are left unfixed). In other words, Chinese readers may need to go back to long sentences to confirm their exact meaning [77].

Eye Movements in Reading Russian

Russian, together with Chinese, is among the five most widely spoken languages in the world and the most widely spoken language using the Cyrillic alphabet. Characteristics of Russian, such as its writing system which is in the middle of the continuum between shallow and deep orthographies with quite complex, but sufficiently regular and predictable phoneme–grapheme correspondences [78], and its rich inflectional and derivational morphology, are of considerable interest for comparative reading research.

Descriptive statistics for basic eye movement variables that are considered as fundamental measures of reading fluency (skipping, first fixation duration, gaze duration, total fixation duration, number of fixations on the word, regression-in, and rereading) for Russian along with 12 other languages is provided in the MECO [47] and MECO Wave 2 [79].

The study by Laurinavichyute and colleagues [23] presents the Russian Sentence Corpus and establishes basic eye movement benchmarks for reading in Russian. It provides descriptive corpus statistics for reading Russian in the form of the average saccade length, landing site, fixation duration measures, and probabilities of skipping and fixating words, as well as proportions of regressions during the reading of natural sentences. It was shown that Russian does not differ from other alphabetic and logographic languages regarding the “big three” variables (word length, frequency, predictability), nor in mean fixation duration and mean saccade amplitude. However, some discrepancies related to the low-level oculomotor characteristics were identified. For example, in Russian, but not in German, the probability of having one fixation on a word increases with the increase in word length and predictability (in Russian, if a word is not fixated once, it is more likely to be skipped than to be fixated more than once, while in German the opposite is true). Given that a prominent characteristic of Russian is its complex morphology, in addition to the “big three” effects, Laurinavichyute and colleagues also account for morphological predictors, namely the part-of-speech category, morphosyntactic ambiguity, and morphological word form (base vs. nonbase). They reported that verb processing requires more effort than noun processing, as reflected in both early (gaze duration) and late (total reading time) measures. Similarly, nonbase wordforms took longer to read than baseword forms; this was also found in both early (first fixation duration) and late (total reading time) measures.

Regarding word length, Alexeeva and Slioussar [80] compared the effect of longer, same-length, and identical parafoveal previews using the gaze-contingent boundary paradigm. They demonstrated that readers of Russian obtain information about word length parafoveally and use it not only to plan subsequent saccades but also for word recognition: first fixation duration and gaze duration were longer in the longer-preview condition than in the same-length condition. Also, the study by Staroverova and colleagues [81] demonstrated that Russian readers rely on orthographic, but not phonological, information extracted from the parafovea.

To sum up, the Chinese language uses the logographic script without apparent word boundaries or apparent distinctions between roots and affixes. In contrast, Russian uses the Cyrillic alphabet, exhibits complex but regular phoneme–grapheme correspondences, and features a rich system of morphosyntactic rules. The differences between the two languages may result in specific processing difficulties for Chinese learners of Russian as well as Russian learners of Chinese [82].

In this study, we aim to investigate the role of universal effects in L2 oculomotor reading behavior, focusing on the typological differences between the readers’ L1 and L2, namely their script and grammar systems. Assuming that reading strategies of L1 affect those of the L2 in non-advanced readers, we hypothesize that native readers of Chinese, which is characterized by visually dense logographic script and monomorphemic word structure, will demonstrate an enhanced word length effect when reading L2 Russian, which has an alphabetic script and a polymorphemic word structure. Furthermore, higher rates of homonymy and homography in monosyllabic tonal Chinese may enhance anticipatory processes during reading. This, in turn, could modulate the word predictability effect not only in L1 Chinese reading (which is beyond the scope of this study) but also in L2 Russian reading. We are particularly interested in the role of these universal effects at different processing stages, as reflected in early and late eye movement measures. Finally, we will investigate the role of language exposure (the duration of studying L2) on the magnitude of these universal effects in L2 reading.

## 2. Materials and Methods

### 2.1. Materials

We developed a corpus for L2 learners of Russian; more detailed information regarding its composition and structure can be found in [83]. The corpus is based on the Russian Sentence Corpus [23], the Russian Child Sentence Corpus [84], and textbooks for the Test of Russian as a Foreign Language [85]. It consists of 90 sentences, all of which contain only words from the vocabulary lists for the B1 and B2 levels of Russian. Half of the sentences had a simple syntactic structure, and the other half were complex. The mean sentence length was 7.6 words, maximum 11 words, and minimum 4 words. Inferential questions were constructed for 30% of the sentences to assess reading comprehension.

For each word in the Corpus, wordform frequency was obtained from the Russian National Corpus (ruscorpora.ru). The criteria for the query were texts which were updated from 1985 and after to reflect frequency in a relatively modern time range. This search yielded 53,526 texts, corresponding to 143,287,761 word tokens. The predictability of each word was assessed in a pretest: a cloze task was used and at least 30 predictions were collected for each word in a sentence [83].

### 2.2. Participants

A total of 41 Chinese-speaking learners of Russian (age range: 19–33 years) and 40 native Russian speakers (age range: 18–30 years) participated in the study on a voluntary basis. All participants had completed higher education or were current university students. All reported normal or corrected-to-normal vision and no history of speech, learning, or hearing disorders. The experiment was conducted in accordance with the Declaration of Helsinki and relevant Russian and international regulations for research ethics. Informed consent was obtained from all participants.

The 41 Chinese participants were recruited through several channels, including university and community advertising. All participants were living in Russia at the time of the experiment. The participants had begun learning Russian either in China or in Russia and had an A2-B2 level of proficiency in Russian at the time of participation. Russian proficiency was assessed in one of two ways: the Test of Russian as a Foreign Language (TORFL) [85] or the Chinese version of the Russian proficiency test. The TORFL is based on a unified certification system for evaluating and certifying Russian language proficiency for non-native speakers. It consists of six levels of increasing difficulty, from Beginner (A1) to Advanced (C2). The test includes five sections that examine a candidate’s proficiency in different language aspects: “Writing”, “Grammar and Vocabulary”, “Reading”, “Listening”, and “Speaking”. The TORFL is an official exam that certifies proficiency levels according to the Common European Framework of Reference (CEFR). The Chinese version is a local equivalent to the TORFL, comprising eight levels. Among the participants in the current study, eight were A2 level, with eleven at B1 and twenty-two at B2. Participants had varying experiences in terms of the duration of studying Russian and time living in Russia (for descriptive statistics, see Table 1). On average, participants had four years of experience living in Russia and four years of learning the Russian language.

### 2.3. Apparatus and Procedure

Eye movements were recorded using an EyeLink 1000+ eye tracker (SR Research, Toronto, ON, Canada). A chinrest was used to minimize head movements. The sampling rate was set at 1000 Hz. Sentences were presented in 18-pt PT Mono font, in black on a gray background. The screen resolution was 1600 × 1024 pixels with a refresh rate of 85 Hz. A 9-point calibration was performed to ensure tracking accuracy, and participants were recalibrated if necessary. The participants were seated at a comfortable distance of 55 cm from the camera and 90 cm from the monitor. Each trial began with a manual drift-correction procedure; a drift-correction point was located slightly to the left of the position of the first word of the following sentence. Calibration was repeated between trials when necessary. Subsequently, a sentence was presented in the center of the screen, with the first word of each sentence aligned to the same starting point.

Participants were instructed to read each sentence carefully and to press the spacebar when finished. Comprehension questions were asked after 30% of the sentences. For each question, three answer options were provided, only one of which was correct. The questions were displayed in the center of the screen, and the answers were given by pressing the corresponding key on the keyboard.

### 2.4. Statistical Analysis

Prior to the analysis of eye-tracking measures, we conducted a standard four-stage fixation cleaning procedure using DataViewer software version 3.1.1 (SR Research). First, we merged all the fixations shorter than 80 ms with adjacent fixations if the distance between them was less than 0.5 angular degrees. Next, we deleted all fixations shorter than 60 ms and longer than 2000 ms. The analysis was conducted on all words in the corpus, excluding the first word of each sentence.

Participants who gave less than 70% correct answers to comprehension questions were excluded from the analysis. For the L2 readers, data from 10 participants were excluded: one at the A2 level, four at the B1 level, and five at the B2 level.

For the analyses, we chose the following measures: gaze duration and first pass skipping rate as the early measures, and rereading rate and regressions-in rate as the late measures. Gaze duration, or first-pass reading time, along with skipping probability, are among the most commonly analyzed early measures in eye-tracking reading research. Regression rates are highly indicative late eye movement measures that reflect contextual integration processes. Although rereading time is less commonly examined than total viewing time, we propose that it is a more precise measure for late effects because, unlike total viewing time, it excludes first-pass reading time from its calculation [86]. The gaze duration analysis included words that were not skipped during the first pass and that had a first fixation duration ranging from 60 ms to 2000 ms. All words were included in the skipping rate analysis. Words for the rereading rate analysis were selected as follows. First, we excluded words that did not elicit any fixations. For the remaining words, we calculated rereading rate as the difference between total viewing time and gaze duration. After that, we excluded words with a rereading time of zero ms, which indicated that these words were not revisited. Finally, we included only those observations with a rereading time of 60 ms or greater. All words were included in the regression-in analysis; for words that did not elicit any fixations, the regression-in value was recorded as zero. The number of observations for each measure is presented in Table A1, Table A2, Table A3, Table A4, Table A5, Table A6, Table A7, Table A8, Table A9, Table A10, Table A11 and Table A12 in Appendix A.

Linear mixed-effects modeling was used to explore relationships between the “big three” variables (word length, wordform frequency, and word predictability) and the continuous measures: gaze duration and rereading time. Generalized linear mixed-effect modeling was used for the binomial measures: skipping and regression-in rates.

First, we ran a model on combined L1 and L2 data with the following fixed effects: wordform frequency, word length, word predictability, participant group (L1 readers and L2 readers), sentence length in words (a control effect), and their interactions: word length and wordform frequency; participant group and wordform frequency; participant group and word length; participant group and predictability; and participant group and sentence length. The random structure comprised participant, sentence, and word intercepts.

We then simplified the model by eliminating non-significant variables to identify the best-fitting model using either step function (in *lmer* analyses) or manual comparison via anova function (in *glmer* analysis).

Subsequently, we constructed separate models for each participant group (L1 readers and L2 readers). The models’ fixed effects included wordform frequency, word length, word predictability, sentence length, and the interaction between wordform frequency and word length. For the L2 group, the models also included the duration of studying Russian (as a measure of language proficiency) and its two-way interactions with wordform frequency, word length, predictability, and sentence length. The random structure was alike as in the combined analysis. As with the full model, non-significant variables were removed from the model.

In all analyses, gaze duration and rereading time were log-transformed to ensure the normal distribution of residuals. Word length and sentence length were scaled, word frequency was log-transformed, and word predictability was logit-transformed [23]. Participant group was orthogonally coded as sum contrast (with L1 readers a baseline).

We ran *(g)lmer* analysis using the *lme4* package [87] in R (version 4.5.1). The *lmerTest* package [88] was used for estimating *p*-values. The plots were generated with *ggplot2* [89] based on partial effects, with variance attributable to random and (controlled) fixed effects using the *keepef* function [90].

## 3. Results

### 3.1. Gaze Duration

**Figure 1:**
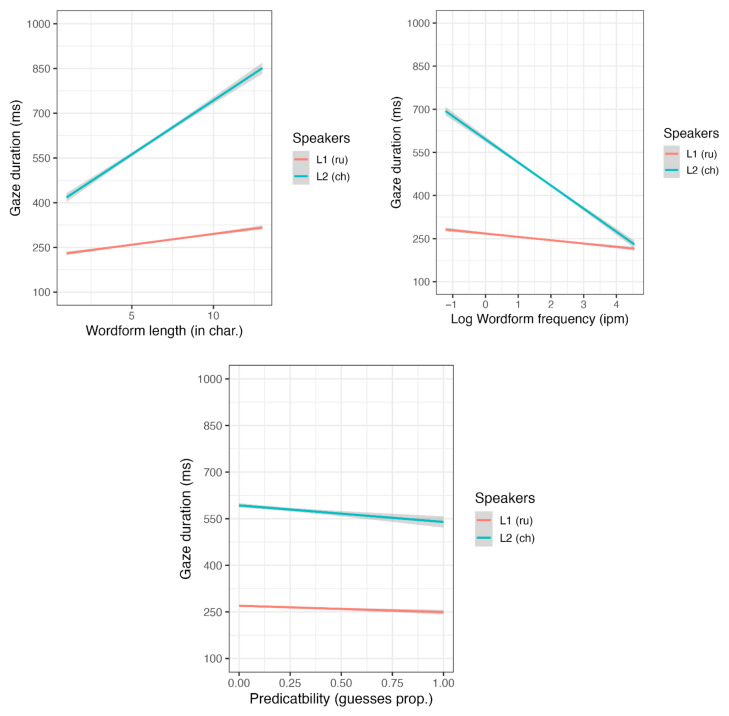
The word length, frequency, and predictability effects on gaze duration.

In the combined analysis (see Appendix A, Table A1), we found significant main effects for word length, wordform frequency, and participant group. As expected, shorter and more frequent words were read faster, and native speakers read more quickly than L2 learners. Our final model excluded sentence length, as this variable did not reach statistical significance. However, the interactions between participant group and word length, participant group and wordform frequency, as well as participant group and predictability, were significant (see Figure 1). Although the main effect of predictability was not significant, L2 readers exhibited slower reading times for longer and less frequent words than L1 readers. It appeared that predictable words sped up word recognition for L2 but not for L1 readers; however, separate analyses (see below) did not confirm this, as the predictability effect was not significant for either L1 or L2 readers.

**Figure 2:**
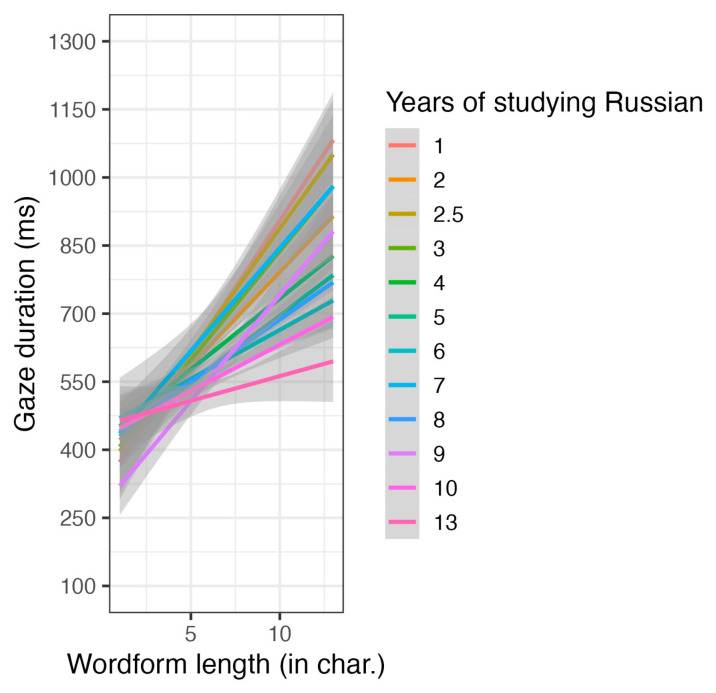
The duration of studying Russian and word length interaction on gaze duration for L2 speakers.

We ran two separate models for L1 and L2 readers (see Appendix A, Table A2 and Table A3) and found significant main effects of word length and wordform frequency in both groups. For L2 readers, the only significant interaction was between the duration of studying Russian and word length (see Figure 2). They read longer words more slowly, particularly in the early stages of learning Russian. However, there was no main effect of the duration of studying Russian on gaze duration itself.

### 3.2. Skipping Rate

We analyzed data from both L1 and L2 reading using a combined model (see Appendix A, Table A4) and found that skipping rates were affected by word length and wordform frequency. Additionally, L1 readers skipped significantly more than L2 learners.

**Figure 3:**
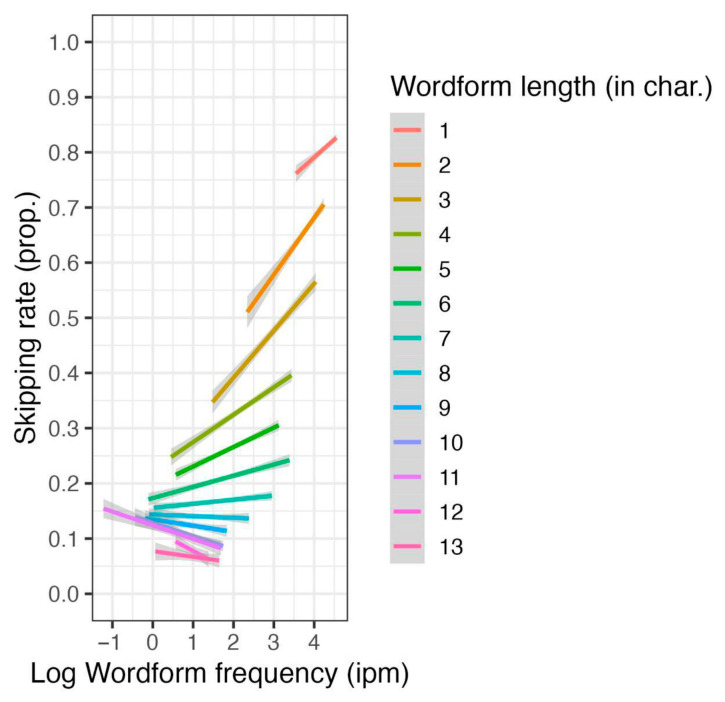
The effect of wordform frequency and word length interaction on skipping rate.

**Figure 4:**
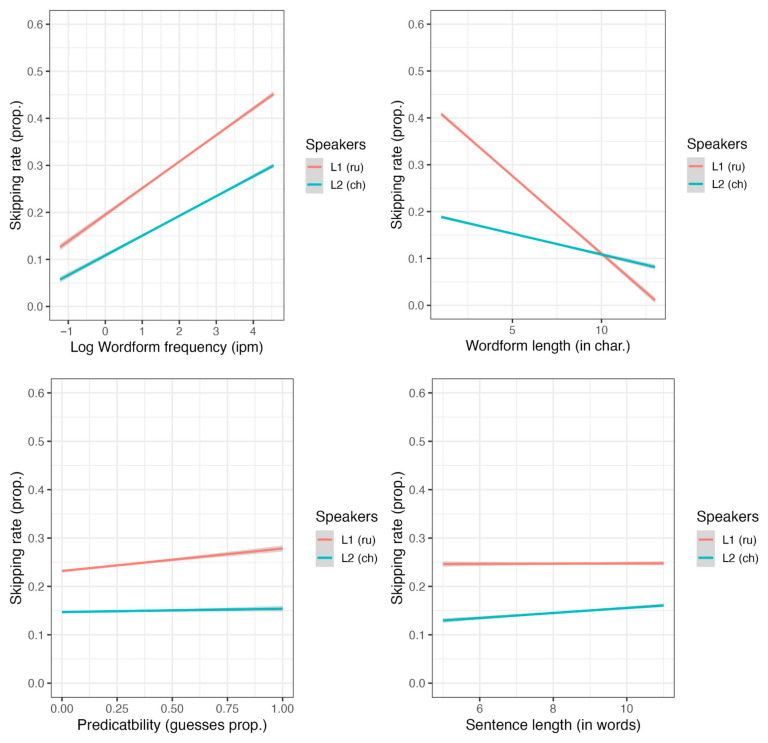
The effects of “big three” (wordform frequency, word length, and predictability) and sentence length on skipping rate.

Length and wordform frequency significantly interacted—short and frequent words were the most probable to be skipped (see Figure 3). The reader group factor (L1 or L2) significantly interacted with word length, wordform frequency, predictability, and sentence length (see Figure 4). Long, low-frequency words were particularly difficult for L2 readers, whereas—as the separate analyses confirmed—predictability had a significance only for L1 readers.

We also created separate models for L1 and L2 readers (see Appendix A, Table A5 and Table A6). In the L1 group, we found significant effects of wordform frequency, word length, and their interaction, as well as a significant effect of predictability. In the L2 group, predictability and word length were not significant, whereas wordform frequency and the interaction between length and frequency were significant. Furthermore, we found no significant main effect of the number of years spent studying Russian, nor any significant interactions involving this variable.

### 3.3. Rereading Rate

**Figure 5:**
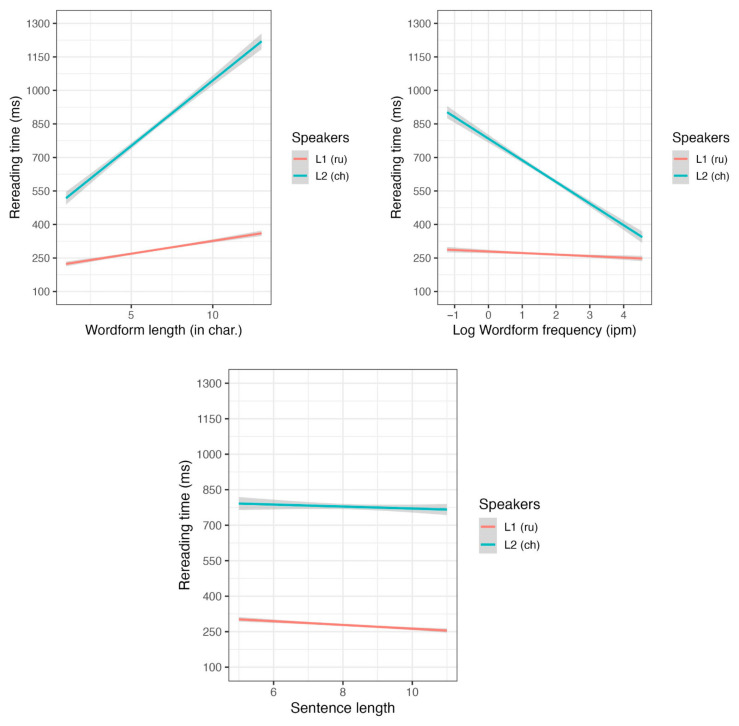
The word length, frequency, and sentence length effects on rereading time.

The combined model revealed significant main effects of the “big three” factors and the “reader group” factor, but not of the sentence length factor (see Appendix A, Table A7). Rereading times were lower for frequent, short, and predictable words, and were also lower for L2 readers than for L1 readers. Word length, wordform frequency, and sentence length significantly interacted with the reader group (see Figure 5). To interpret the significant interactions, we conducted separate analyses.

In the L1 group (see Appendix A, Table A8), we found significant effects of word length and sentence length on rereading time, with shorter words and words in longer sentences requiring less rereading time. For L2 readers (see Appendix A, Table A9), the “big three” effects were significant. However, we found no significant main effect of the duration of studying Russian, nor any interactions involving this variable.

Based on the significant interactions from the combined analyses, we can conclude that longer words slowed down L2 readers to a greater extent. The main effect of wordform frequency was significant in the combined analysis; however, it may be attributed to L2 readers, as they slowed down more for less frequent words. In the separate model for L1 readers, the effect of wordform frequency did not reach significance. The same pattern was observed for word predictability: it was significant for L2 readers but not for L1 readers. Conversely, the significance effect of sentence length was due to L1 readers, as it was not significant for L2 readers in their separate model.

### 3.4. Regression in Rates

**Figure 6:**
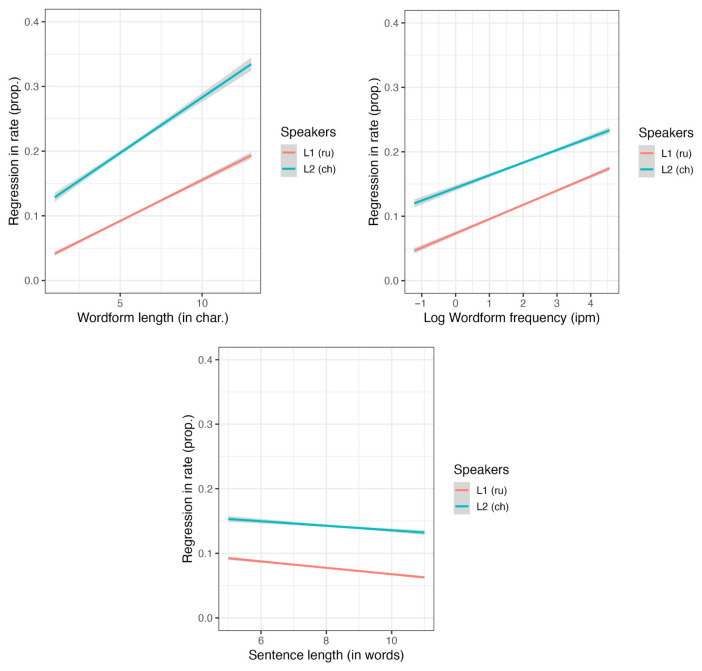
The effects of wordform frequency, length and sentence length on regression in rate.

We analyzed data from both L1 and L2 reading using a combined model and found that all the “big three” factors significantly influenced regression rates (see Appendix A, Table A10). Notably, the effect of wordform frequency was in the opposite direction that expected: regression rates were higher for more frequent wordforms. Additionally, L2 readers exhibited significantly more regressions than L1 readers. The effects of word length, wordform frequency, and sentence length significantly interacted with the reader group (see Figure 6).

**Figure 7:**
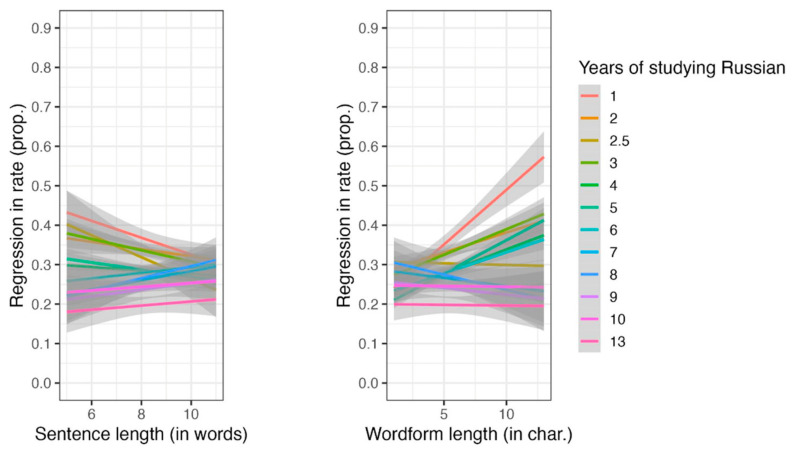
The interactions between word length and the duration of studying Russian, and sentence length and the duration of studying Russian.

Separate models for L1 readers revealed significant effects of word predictability and word length (see Appendix A, Table A11). For L2 readers, there was a significant main effect of word predictability, along with interactions between word length and the duration of studying Russian, and between sentence length and the duration of studying Russian (see Figure 7 and Appendix A, Table A12). However, the main effects of word length, sentence length, and the duration of studying Russian were not significant. These results suggest that the effects of word length and sentence length become less pronounced as the duration of studying Russian increases.

The effect of wordform frequency did not reach significance in either of the separate models. Therefore, its presence in the combined model for L1 and L2 readers is somewhat artificial. The significant interaction between group and word length indicated that longer words posed greater challenges for L2 readers, leading to more regressions. This interaction between group and sentence length was significant in the combined model. The effect was primarily driven by the performance of L2 readers, as no such effect was observed among L1 readers.

### 3.5. Results Sum-Up

Regarding gaze duration, we found significant effects of wordform frequency and word length for both L1 and L2 readers. These effects were more pronounced in L2 learners. It should be noted that L2 readers read longer words more slowly, particularly during the early stages of studying Russian.

Regarding skipping rates, we found that all the “big three” effects significantly influenced skipping rates in L1 readers. In contrast, for L2 readers, only wordform frequency and word length were significant; predictability was not. L1 readers skipped highly predictable words, but this factor was not significant for L2 readers.

Both L1 and L2 readers spent more time rereading longer words, with this effect being more pronounced in L2 readers. Furthermore, rereading time in L2 readers was also influenced by wordform frequency and word predictability. For L1 readers, however, rereading time was influenced by the sentence length.

Although wordform frequency did not significantly impact regression rate in either L1 and L2 readers, the interaction between reader group and word length indicated that longer words presented greater difficulties for L2 readers, resulting in increased regressions. This effect was absent in L1 readers, indicating a distinct challenge faced by L2 learners. Additionally, the analysis suggested that as L2 readers gained more years of study of Russian, the influence of wordform and sentence length on their reading performance diminished.

## 4. Discussion

Our study aimed to present eye movement data for a typologically different L1/L2 pair—native Russian L1 readers and Chinese L2 readers of Russian—and to explore the effect of the “big three” factors (wordform frequency, word length, and predictability) on early and late eye movement measures. We demonstrated that wordform frequency, word length, and word predictability significantly influence both early and late eye movement measures for L1 and L2 readers of Russian. Furthermore, we found that the size of each effect was much larger for L2 readers than for L1 readers.

### 4.1. Word Length Effect

Our study revealed a word length effect on both early (gaze duration, skipping rate) and late (rereading time, regression rate) eye movement measures in both native and non-native readers. This demonstrates the significant influence of word length on all readers and across all oculomotor metrics. Word length appears to be an important factor at all stages of the reading process, from decoding and lexical access to integration. These results align with the findings of Siegelman and colleagues [47], who showed that differences in oculomotor reading behavior across languages, particularly the variability in skipping rates, can be largely explained by cross-linguistic differences in word length distributions.

Although the crucial role of word length has been previously demonstrated in multiple L2 reading studies, we also show that for L2 readers word length is the only factor that interacts significantly with the duration of studying L2, which is a novel finding. It highlights the process of adaptation of native Chinese speakers to alphabetic script and polymorphemic word structure at the upper-elementary to intermediate level. Mashanlo [82], who investigated the oculomotor reading behavior of both Chinese L2 learners of Russian and Russian L2 learners of Chinese, also reports that increased L2 exposure leads to shorter fixation durations in both groups which can be explained by their adaptation to a non-native script. A question for further research is to investigate the perceptual span of L2 readers and the capacity for parafoveal processing (see Fernandez and Allen [62]) which is especially interesting in the case of different-script bilinguals.

### 4.2. Wordform Frequency Effect

We demonstrate an effect of wordform frequency on early eye movement measures (gaze duration, skipping rate) for both L1 and L2 readers groups, as frequency plays a crucial role in initial stages of reading, namely visual word recognition and lexical access. However, for late eye movement measures (rereading time), wordform frequency was significant only for L2 readers. They are not only slowed down by low-frequency words during first-pass reading but also experienced difficulties with semantic and syntactic integration in later processing stages. Although all the words in the corpus were included in the vocabulary lists for the B1 and B2 levels, less frequent words could be unfamiliar to L2 learners which could impede contextual integration. In contrast, native readers at the later processing stages, when the word meaning is already retrieved from mental lexicon, were influenced by contextual factors, such as word predictability and sentence length. This result aligns with previous work on frequency effect and language experience by Berzak and Levy [49] which showed that a lack of language experience results in the frequency effect on late eye movement measures. Also, the typologically distant L1 background rules out any cognates which could influence lexical access (see [91,92]).

### 4.3. Word Predictability Effect

Our findings indicate that word predictability influences early eye movement measures, namely the skipping rate, in L1 readers but not in L2 readers. For native speakers, the influence of predictability on eye movements is immediate: prior to fixating on a word, they use context and parafoveal processing to anticipate it and subsequently decide whether to skip it. In contrast, L2 readers do not exhibit this effect in early measures; due to limited language exposure, they lack the capacity for rapid anticipation of upcoming words. Consequently, predictability does not affect their probability of skipping. This observation aligns with the idea of lexicon–context tradeoff [21,49], which suggests that readers with limited language experience tend to rely more on stable word properties, such as frequency, rather than context-dependent factors. Also, a possible explanation of this finding is the reduced parafoveal preview benefit which results in a slower pace of semantic processing [62,63]. We show that predictability for L2 readers becomes significant in late measures, as they spend more time rereading words in less predictable contexts. For L1 readers, predictability also affects late eye movement measures, specifically regression rates, but not rereading time.

Thus, for L2 readers compared to L1 readers, the frequency effect appears to be extended, persisting even after the initial reading of a word, as evidenced by both early and late eye movements. In contrast, the predictability effect is delayed for L2 readers, manifesting only in later measures after the initial word has been processed and attention has shifted. This indicates that predictability becomes relevant for L2 readers only during later integration processes. Native speakers, however, exhibit the predictability effect in both early and late measures.

## 5. Conclusions

Our study demonstrated that the “big three” effects significantly influence eye movements in both L1 and L2 readers, but their impact on early and late eye movement measures differs between groups. We identified robust early and late effects of word length in both L1 and L2 readers of Russian, a language characterized by long, polymorphemic words. Notably, native Chinese speakers learning Russian as L2 exhibited increasing adaptation to the alphabetic system as their years of study increased. This finding underscores the challenges involved in adjusting to a different writing system.

In L1 readers, wordform frequency primarily influenced early processing stages, whereas in L2 readers, it remained a critical factor during later stages as well. We also observed a lexicon–context tradeoff in Chinese learners of L2 Russian, a finding that contradicts our hypothesis that these readers would demonstrate an enhanced predictability effect in their L2 Russian reading. This suggests that the participants’ proficiency level may not have reached the threshold required to rely on contextual predictability for achieving native-like reading fluency. At the A2-B2 proficiency levels, they predominantly navigate text based on their expectations of wordform frequency.

These findings contribute to the existing knowledge on the interaction between two languages that differ significantly in their writing systems and grammatical structures. Furthermore, they provide new insights into the specific processing challenges encountered by Chinese learners of Russian. The corpus is freely accessible at the OSF and can serve as a valuable resource for further research on the cognitive processes involved in L2 reading.

It is important to acknowledge that the Russian language proficiency level of the native Chinese speakers in our sample was not advanced. Consequently, the eye movement data may have been significantly influenced by varying proficiency levels, which limits the implications of our discussion and conclusions.

Additionally, our research is constrained by a lack of data regarding the daily usage of the Russian language among non-native speakers. Information regarding language exposure—specifically, how frequently learners used the language in everyday contexts outside the classroom—would provide valuable insights into our findings. It is possible that not only the duration of Russian language study interacts with the word length effect but also that language exposure influences wordform frequency and contextual predictability.

Future research could benefit from examining a broader range of non-native speakers and incorporating additional languages for comparative analysis, thereby enhancing our understanding of the reading development processes in L2 learners. Furthermore, the word predictability and frequency data collected from the L2 learners may lead to new insights of the effects of these parameters on eye movement measures. High-frequency and high-predictable words for Russian native speakers are not necessarily the same for L2 learners.

## Figures and Tables

**Table 1:** Data of language experience and years of studying Russian.

Participant’s Experience	Mean, SD (Years)
Duration of studying Russian language	4.67 (2.71)
Time of living in Russia	4.16 (2.70)

Note. The table presents the mean and standard deviation (SD) for the duration of Russian language study and the time spent living in Russia among the Chinese-speaking learners of Russian.

## Data Availability

The Corpus materials are available through the following link: https://osf.io/2kdu3/?view_only=3a488dc691b74a9b97e9ea0c61c063fd (accessed on 6 October 2025). The data and analysis code for this study can be obtained by contacting the corresponding author.

## Appendix A

**Table A1:** Linear mixed-effects model outputs for combined model (L1 and L2 readers) for gaze duration results.

	Gaze Duration (ms)			
Coefficient	Estimates	Std. Error	Conf. Int (95%)	*p*-Value
(Intercept)	5.84	0.03	5.78–5.90	<0.001 ***
Log wordform frequency (LWF)	−0.09	0.01	−0.12–−0.07	<0.001 ***
Logit word predictability (LWP)	−0.01	0.01	−0.02–0.00	0.198
Scaled word length (SWL)	0.09	0.01	0.07–0.12	<0.001 ***
Reader group—L2 (Group)	0.38	0.02	0.34–0.42	<0.001 ***
LWP × Group	0.01	0.00	0.00–0.01	0.042 *
SWL × Group	0.03	0.00	0.02–0.04	<0.001 ***
LWF × Group	−0.06	0.00	−0.06–−0.05	<0.001 ***
**Random Effects**				
σ^2^	0.24			
τ_00word.id_	0.03			
τ_00item.id_	0.00			
τ_00subj_	0.02			
ICC	0.16			
N_subj_	71			
N_item.id_	89			
N_word.id_	477			
Observations	29,816			
Marginal R^2^/Conditional R^2^	0.271/0.389			

Note. We built a linear mixed-effects model to investigate the gaze duration for L1 and L2 readers. The combined model was built with the following fixed effects: word frequency, word length, word predictability, participant group (L1 readers and L2 readers), sentence length in words (a control effect), and their interactions: word length and frequency; participant group and word frequency; participant group and word length; participant group and predictability; and participant group and sentence length. The random structure comprised participant, sentence, and word intercepts. The simplified model with eliminating non-significant variables presented in the table. * *p* < 0.05; *** *p* < 0.001.

**Table A2:** Linear mixed-effects model outputs for model L1 readers for gaze duration results.

	L1: Gaze Duration (ms)			
Coefficient	Estimates	Std. Error	Conf. Int (95%)	*p*-Value
(Intercept)	5.48	0.03	5.42–5.54	<0.001 ***
Log wordform frequency (LWF)	−0.04	0.01	−0.06–−0.01	0.003 **
Logit word predictability (LWP)	0.06	0.01	0.03–0.08	<0.001 ***
**Random Effects**				
σ^2^	0.20			
τ00word.id	0.03			
τ00subj	0.02			
ICC	0.19			
Nsubj	40			
Nword.id	477			
Observations	15,983			
Marginal R^2^/Conditional R^2^	0.026/0.207			

Note. We built a linear mixed-effects model to investigate the gaze duration for L1 readers. The model’s fixed effects included word frequency, word length, predictability, sentence length, and the interaction between word frequency and word length. The random structure comprised participant, sentence, and word intercepts. The simplified model with eliminating non-significant variables presented in the table. ** *p* < 0.01; *** *p* < 0.001.

**Table A3:** Linear mixed-effects model outputs for model L2 readers for gaze duration results.

	L2: Gaze Duration (ms)			
Coefficient	Estimates	Std. Error	Conf. Int (95%)	*p*-Value
(Intercept)	6.23	0.04	6.16–6.31	<0.001 ***
Log wordform frequency (LWF)	−0.16	0.01	−0.18–−0.13	<0.001 ***
Scaled word length (SWL)	0.12	0.01	0.09–0.15	<0.001 ***
Years of studying Russian (YSR)	−0.04	0.03	−0.10–0.02	0.173
SWL × YSR	−0.02	0.01	−0.03–−0.01	<0.001 ***
**Random Effects**				
σ^2^	0.28			
τ00word.id	0.03			
τ00subj	0.03			
ICC	0.17			
Nsubj	31			
Nword.id	477			
Observations	13,833			
Marginal R^2^/Conditional R^2^	0.160/0.304			

Note. We built a linear mixed-effects model to investigate the gaze duration for L2 readers. The model’s fixed effects included word frequency, word length, predictability, sentence length, and the interaction between word frequency and word length as well as duration of studying Russian (as a measure of language proficiency) and its four two-interactions with word frequency, word length, predictability, and sentence length. The random structure comprised participant, sentence, and word intercepts. The simplified model with eliminating non-significant variables presented in the table. *** *p* < 0.001.

**Table A4:** Linear mixed-effects model outputs for combined model (L1 and L2 readers) for skipping rate results.

	Skipping Rate			
Coefficient	Odds Ratios	Std. Error	Conf. Int (95%)	*p*-Value
(Intercept)	0.20	0.02	0.16–0.25	<0.001 ***
Log wordform frequency (LWF)	1.20	0.04	1.13–1.28	<0.001 ***
Logit word predictability (LWP)	1.02	0.02	0.98–1.06	0.418
Scaled word length (SWL)	0.78	0.04	0.70–0.86	<0.001 ***
Scaled sentence length (SSL)	1.01	0.03	0.96–1.07	0.641
Reader group—L2 (Group)	0.70	0.06	0.59–0.82	<0.001 ***
LWF × SWL	0.76	0.02	0.72–0.79	<0.001 ***
LWP × Group	0.96	0.01	0.93–0.98	0.002 **
SWL × Group	1.18	0.03	1.13–1.23	<0.001 ***
LWF × Group	1.08	0.02	1.05–1.11	<0.001 ***
SSL × Group	1.03	0.01	1.01–1.06	0.007 **
**Random Effects**				
σ^2^	3.29			
τ_00word.id_	0.14			
τ_00item.id_	0.03			
τ_00subj_	0.41			
ICC	0.15			
N_subj_	71			
N_item.id_	89			
N_word.id_	477			
Observations	44,642			
Marginal R^2^/Conditional R^2^	0.270/0.380			

Note. We built a linear mixed-effects model to investigate the gaze duration for L1 and L2 readers. The combined model was built with the following fixed effects: word frequency, word length, word predictability, participant group (L1 readers and L2 readers), sentence length in words (a control effect), and their interactions: word length and frequency; participant group and word frequency; participant group and word length; participant group and predictability; and participant group and sentence length. The random structure comprised participant, sentence, and word intercepts. The simplified model with eliminating non-significant variables presented in the table. ** *p* < 0.01, *** *p* < 0.001.

**Table A5:** Linear mixed-effects model outputs for model L1 readers for skipping rate results.

	L1: Skipping Rate			
Coefficient	Odds Ratios	Std. Error	Conf. Int (95%)	*p*-Value
(Intercept)	0.27	0.04	0.21–0.35	<0.001 ***
Log wordform frequency (LWF)	1.14	0.05	1.05–1.24	0.003 **
Logit word predictability (LWP)	1.07	0.03	1.01–1.12	0.014 *
Scaled word length (SWL)	0.66	0.05	0.57–0.75	<0.001 ***
LWL × SWL	0.76	0.02	0.71–0.81	<0.001 ***
**Random Effects**				
σ^2^	3.29			
τ_00word.id_	0.27			
τ_00item.id_	0.03			
τ_00subj_	0.38			
ICC	0.17			
N_subj_	40			
N_item.id_	89			
N_word.id_	477			
Observations	25,160			
Marginal R^2^/Conditional R^2^	0.294/0.414			

Note. We built a linear mixed-effects model to investigate the gaze duration for L1 readers. The model’s fixed effects included word frequency, word length, predictability, sentence length, and the interaction between word frequency and word length. The random structure comprised participant, sentence, and word intercepts. The simplified model with eliminating non-significant variables presented in the table. * *p* < 0.05; ** *p* < 0.01; *** *p* < 0.001.

**Table A6:** Linear mixed-effects model outputs for model L2 readers for skipping rate results.

	L2: Skipping Rate			
Coefficient	Odds Ratios	Std. Error	Conf. Int (95%)	*p*-Value
(Intercept)	0.15	0.02	0.11–0.20	<0.001 ***
Log wordform frequency (LWF)	1.24	0.04	1.16–1.32	<0.001 ***
Scaled word length (SWL)	0.94	0.05	0.84–1.05	0.258
LWL × SWL	0.74	0.02	0.70–0.77	<0.001 ***
**Random Effects**				
σ^2^	3.29			
τ_00word.id_	0.05			
τ_00item.id_	0.07			
τ_00subj_	0.48			
ICC	0.15			
N_subj_	31			
N_item.id_	89			
N_word.id_	477			
Observations	19,482			
Marginal R^2^/Conditional R^2^	0.236/0.354			

Note. We built a linear mixed-effects model to investigate the gaze duration for L2 readers. The model’s fixed effects included word frequency, word length, predictability, sentence length, and the interaction between word frequency and word length as well as duration of studying Russian (as a measure of language proficiency) and its four two-interactions with word frequency, word length, predictability, and sentence length. The random structure comprised participant, sentence, and word intercepts. The simplified model with eliminating non-significant variables presented in the table. *** *p* < 0.001.

**Table A7:** Linear mixed-effects model outputs for combined model (L1 and L2 readers) for reading time results.

	Rereading Time (ms)			
Coefficient	Estimates	Std. Error	Conf. Int (95%)	*p*-Value
(Intercept)	5.95	0.04	5.86–6.04	<0.001 ***
Log wordform frequency (LWF)	−0.08	0.02	−0.11–−0.05	<0.001 ***
Logit word predictability (LWP)	−0.03	0.01	−0.06–−0.01	0.001 ***
Scaled word length (SWL)	0.14	0.02	0.10–0.17	<0.001 ***
Scaled sentence length (SSL)	−0.02	0.02	−0.05–0.01	0.240
Reader group—L2 (Group)	0.50	0.03	0.44–0.55	<0.001 ***
SWL × Group	0.04	0.01	0.02–0.05	<0.001 ***
LWF × Group	−0.06	0.01	−0.08–−0.05	<0.001 ***
SSL × Group	0.02	0.01	0.01–0.03	<0.001 ***
**Random Effects**				
σ^2^	0.39			
τ_00word.id_	0.04			
τ_00item.id_	0.02			
τ_00subj_	0.05			
ICC	0.21			
N_subj_	71			
N_item.id_	89			
N_word.id_	476			
Observations	16,637			
Marginal R^2^/Conditional R^2^	0.284/0.436			

Note. We built a linear mixed-effects model to investigate the gaze duration for L1 and L2 readers. The combined model was built with the following fixed effects: word frequency, word length, word predictability, participant group (L1 readers and L2 readers), sentence length in words (a control effect), and their interactions: word length and frequency; participant group and word frequency; participant group and word length; participant group and predictability; and participant group and sentence length. The random structure comprised participant, sentence, and word intercepts. The simplified model with eliminating non-significant variables presented in the table. *** *p* < 0.001.

**Table A8:** Linear mixed-effects model outputs for model L1 readers for reading time results.

	L1: Rereading Time (ms)			
Coefficient	Estimates	Std. Error	Conf. Int (95%)	*p*-Value
(Intercept)	5.48	0.03	5.41–5.55	<0.001 ***
Scaled word length (SWL)	0.10	0.01	0.07–0.13	<0.001 ***
Scaled sentence length (SSL)	−0.04	0.02	−0.07–−0.01	0.011 *
**Random Effects**				
σ^2^	0.32			
τ_00word.id_	0.03			
τ_00item.id_	0.01			
τ_00subj_	0.04			
ICC	0.21			
N_subj_	40			
N_item.id_	89			
N_word.id_	472			
Observations	7410			
Marginal R^2^/Conditional R^2^	0.025/0.225			

Note. We built a linear mixed-effects model to investigate the gaze duration for L1 readers. The model’s fixed effects included word frequency, word length, predictability, sentence length, and the interaction between word frequency and word length. The random structure comprised participant, sentence, and word intercepts. The simplified model with eliminating non-significant variables presented in the table. * *p* < 0.05; *** *p* < 0.001.

**Table A9:** Linear mixed-effects model outputs for model L2 readers for reading time results.

	L2: Rereading Time (ms)			
Coefficient	Estimates	Std. Error	Conf. Int (95%)	*p*-Value
(Intercept)	6.43	0.06	6.31–6.56	<0.001 ***
Log wordform frequency (LWF)	−0.14	0.02	−0.18–−0.11	<0.001 ***
Logit word predictability (LWP)	−0.04	0.01	−0.07–−0.02	0.002 **
Scaled word length (SWL)	0.17	0.02	0.13–0.21	<0.001 ***
**Random Effects**				
σ^2^	0.43			
τ_00word.id_	0.05			
τ_00item.id_	0.03			
τ_00subj_	0.06			
ICC	0.25			
N_subj_	31			
N_item.id_	89			
N_word.id_	476			
Observations	9227			
Marginal R^2^/Conditional R^2^	0.118/0.339			

Note. We built a linear mixed-effects model to investigate the gaze duration for L2 readers. The model’s fixed effects included word frequency, word length, predictability, sentence length, and the interaction between word frequency and word length as well as duration of studying Russian (as a measure of language proficiency) and its four two-interactions with word frequency, word length, predictability, and sentence length. The random structure comprised participant, sentence, and word intercepts. The simplified model with eliminating non-significant variables presented in the table. ** *p* < 0.01; *** *p* < 0.001.

**Table A10:** Linear mixed-effects model outputs for combined model (L1 and L2 readers) for regression in rate results.

	Regressions-in Rate			
Coefficient	Odds Ratios	Std. Error	Conf. Int (95%)	*p*-Value
(Intercept)	0.09	0.02	0.06–0.15	<0.001 ***
Log wordform frequency (LWF)	1.25	0.14	1.00–1.56	0.047 *
Logit word predictability (LWP)	0.70	0.03	0.65–0.76	<0.001 ***
Scaled word length (SWL)	1.32	0.16	1.05–1.67	0.017 *
Scaled sentence length (SSL)	0.92	0.06	0.81–1.05	0.211
Reader group—L2 (Group)	1.44	0.12	1.22–1.70	<0.001 ***
SWL × Group	0.95	0.02	0.91–0.99	0.010 *
LWF × Group	0.96	0.02	0.93–0.99	0.010 *
SSL × Group	1.03	0.01	1.00–1.05	0.038 *
**Random Effects**				
σ^2^	3.29			
τ_00word.id_	2.72			
τ_00item.id_	0.24			
τ_00subj_	0.40			
ICC	0.51			
N_subj_	71			
N_item.id_	89			
N_word.id_	477			
Observations	44,642			
Marginal R^2^/Conditional R^2^	0.032/0.521			

Note. We built a linear mixed-effects model to investigate the gaze duration for L1 and L2 readers. The combined model was built with the following fixed effects: word frequency, word length, word predictability, participant group (L1 readers and L2 readers), sentence length in words (a control effect), and their interactions: word length and frequency; participant group and word frequency; participant group and word length; participant group and predictability; and participant group and sentence length. The random structure comprised participant, sentence, and word intercepts. The simplified model with eliminating non-significant variables presented in the table. * *p* < 0.05; *** *p* < 0.001.

**Table A11:** Linear mixed-effects model outputs for model L1 readers for regression in rate results.

	L1: Regressions-in Rate			
Coefficient	Odds Ratios	Std. Error	Conf. Int (95%)	*p*-Value
(Intercept)	0.13	0.02	0.10–0.17	<0.001 ***
Logit word predictability (LWP)	0.78	0.04	0.71–0.86	<0.001 ***
Scaled word length (SWL)	1.17	0.09	1.00–1.37	0.045 *
**Random Effects**				
σ^2^	3.29			
τ_00word.id_	1.98			
τ_00item.id_	0.17			
τ_00subj_	0.43			
ICC	0.44			
N_subj_	40			
N_item.id_	89			
N_word.id_	477			
Observations	25,160			
Marginal R^2^/Conditional R^2^	0.015/0.448			

Note. We built a linear mixed-effects model to investigate the gaze duration for L1 readers. The model’s fixed effects included word frequency, word length, predictability, sentence length, and the interaction between word frequency and word length. The random structure comprised participant, sentence, and word intercepts. The simplified model with eliminating non-significant variables presented in the table. * *p* < 0.05; *** *p* < 0.001.

**Table A12:** Linear mixed-effects model outputs for model L2 readers for regression in rate results.

	L2: Regressions-in Rate			
Coefficient	Odds Ratios	Std. Error	Conf. Int (95%)	*p*-Value
(Intercept)	0.19	0.03	0.15–0.26	<0.001 ***
Logit word predictability (LWP)	0.69	0.03	0.62–0.75	<0.001 ***
Scaled word length (SWL)	1.10	0.09	0.93–1.29	0.261
Scaled sentence length (SSL)	0.97	0.06	0.86–1.08	0.546
Years of studying Russian (YSR)	0.89	0.10	0.72–1.10	0.284
SWL × YSR	0.95	0.02	0.91–0.99	0.008 **
SSL × YSR	1.04	0.02	1.00–1.08	0.050 *
**Random Effects**				
σ^2^	3.29			
τ_00word.id_	2.16			
τ_00item.id_	0.15			
τ_00subj_	0.36			
ICC	0.45			
N_subj_	31			
N_item.id_	89			
N_word.id_	477			
Observations	19,482			
Marginal R^2^/Conditional R^2^	0.025/0.462			

Note. We built a linear mixed-effects model to investigate the gaze duration for L2 readers. The model’s fixed effects included word frequency, word length, predictability, sentence length, and the interaction between word frequency and word length as well as duration of studying Russian (as a measure of language proficiency) and its four two-interactions with word frequency, word length, predictability, and sentence length. The random structure comprised participant, sentence, and word intercepts. The simplified model with eliminating non-significant variables presented in the table. * *p* < 0.05; ** *p* < 0.01; *** *p* < 0.001.

## References

[1] Acha J., Carreiras M. (2014). Exploring the Mental Lexicon: A Methodological Approach to Understanding How Printed Words Are Represented in Our Minds. Ment. Lex..

[2] Staub A. (2015). The Effect of Lexical Predictability on Eye Movements in Reading: Critical Review and Theoretical Interpretation. Lang. Linguist. Compass.

[3] Budiu R. (2004). Interpretation-Based Processing: A Unified Theory of Semantic Sentence Comprehension. Cogn. Sci..

[4] Clifton C., Staub A., Rayner K. (2007). Eye Movements in Reading Words and Sentences. Eye Movements: A Window on Mind and Brain.

[5] Boston M.F., Hale J., Kliegl R., Patil U., Vasishth S. (2008). Parsing Costs as Predictors of Reading Difficulty: An Evaluation Using the Potsdam Sentence Corpus. J. Eye Mov. Res..

[6] Vasishth S., Von Der Malsburg T., Engelmann F. (2013). What Eye Movements Can Tell Us about Sentence Comprehension. WIRES Cogn. Sci..

[7] Rayner K. (1998). Eye Movements in Reading and Information Processing: 20 Years of Research. Psychol. Bull..

[8] Rayner K., Fischer M.H., Pollatsek A. (1998). Unspaced Text Interferes with Both Word Identification and Eye Movement Control. Vis. Res..

[9] Kliegl R., Grabner E., Rolfs M., Engbert R. (2004). Length, Frequency, and Predictability Effects of Words on Eye Movements in Reading. Eur. J. Cogn. Psychol..

[10] Calvo M.G., Meseguer E. (2002). Eye Movements and Processing Stages in Reading: Relative Contribution of Visual, Lexical, and Contextual Factors. Span. J. Psychol..

[11] Rayner K., Fischer M.H. (1996). Mindless Reading Revisited: Eye Movements during Reading and Scanning Are Different. Percept. Psychophys..

[12] Hyönä J., Olson R.K. (1995). Eye Fixation Patterns among Dyslexic and Normal Readers: Effects of Word Length and Word Frequency. J. Exp. Psychol. Learn. Mem. Cogn..

[13] Just M.A., Carpenter P.A. (1980). A Theory of Reading: From Eye Fixations to Comprehension. Psychol. Rev..

[14] Rayner K., Sereno S.C., Raney G.E. (1996). Eye Movement Control in Reading: A Comparison of Two Types of Models. J. Exp. Psychol. Hum. Percept. Perform..

[15] Lee Y.-A., Binder K.S., Kim J.-O., Pollatsek A., Rayner K. (1999). Activation of Phonological Codes during Eye Fixations in Reading. J. Exp. Psychol. Hum. Percept. Perform..

[16] Lima S.D., Pollatsek A. (1983). Lexical Access via an Orthographic Code? The Basic Orthographic Syllabic Structure (BOSS) Reconsidered. J. Verbal Learn. Verbal Behav..

[17] Spoehr K.T., Smith E.E. (1973). The Role of Syllables in Perceptual Processing. Cogn. Psychol..

[18] Prinzmetal W., Presti D.E., Posner M.I. (1986). Does Attention Affect Visual Feature Integration?. J. Exp. Psychol. Hum. Percept. Perform..

[19] Franck J., Millotte S., Posada A., Rizzi L. (2013). Abstract Knowledge of Word Order by 19 Months: An Eye-Tracking Study. Appl. Psycholinguist..

[20] Luke S.G., Christianson K. (2018). The Provo Corpus: A Large Eye-Tracking Corpus with Predictability Norms. Behav. Res. Methods.

[21] Cop U., Drieghe D., Duyck W. (2015). Eye Movement Patterns in Natural Reading: A Comparison of Monolingual and Bilingual Reading of a Novel. PLoS ONE.

[22] Pynte J., Kennedy A. (2006). An Influence over Eye Movements in Reading Exerted from beyond the Level of the Word: Evidence from Reading English and French. Vis. Res..

[23] Laurinavichyute A.K., Sekerina I.A., Alexeeva S., Bagdasaryan K., Kliegl R. (2019). Russian Sentence Corpus: Benchmark Measures of Eye Movements in Reading in Russian. Behav. Res. Methods.

[24] Lavidor M., Whitney C. (2005). Word Length Effects in Hebrew. Cogn. Brain Res..

[25] Ganayim D. (2015). Optimal viewing position effect of connecting and un-connecting letters within letter-string in Arabic. Rom. J. Exp. Appl. Psychol..

[26] Sainio M., Hyönä J., Bingushi K., Bertram R. (2007). The Role of Interword Spacing in Reading Japanese: An Eye Movement Study. Vis. Res..

[27] Pan J., Yan M., Richter E.M., Shu H., Kliegl R. (2021). The Beijing Sentence Corpus: A Chinese Sentence Corpus with Eye Movement Data and Predictability Norms. Behav. Res. Methods.

[28] Rayner K., Duffy S.A. (1986). Lexical Complexity and Fixation Times in Reading: Effects of Word Frequency, Verb Complexity, and Lexical Ambiguity. Mem. Cogn..

[29] Inhoff A.W., Rayner K. (1986). Parafoveal Word Processing during Eye Fixations in Reading: Effects of Word Frequency. Percept. Psychophys..

[30] Juhasz B.J., Rayner K. (2003). Investigating the Effects of a Set of Intercorrelated Variables on Eye Fixation Durations in Reading. J. Exp. Psychol. Learn. Mem. Cogn..

[31] Juhasz B.J., Rayner K. (2006). The Role of Age of Acquisition and Word Frequency in Reading: Evidence from Eye Fixation Durations. Vis. Cogn..

[32] Gerth S., Festman J. (2021). Reading Development, Word Length and Frequency Effects: An Eye-Tracking Study with Slow and Fast Readers. Front. Commun..

[33] Cop U., Dirix N., Drieghe D., Duyck W. (2017). Presenting GECO: An Eyetracking Corpus of Monolingual and Bilingual Sentence Reading. Behav. Res. Methods.

[34] Kim S.Y., Donald J.B. (2017). Effects of Visual, Lexical, and Contextual Factors on Word Recognition in Reading Korean Sentences. J. Cogn. Sci..

[35] Yan G., Tian H., Bai X., Rayner K. (2006). The Effect of Word and Character Frequency on the Eye Movements of Chinese Readers. Br. J. Psychol..

[36] Ehrlich S.F., Rayner K. (1981). Contextual Effects on Word Perception and Eye Movements during Reading. J. Verbal Learn. Verbal Behav..

[37] Smith N.J., Levy R. (2013). The Effect of Word Predictability on Reading Time Is Logarithmic. Cognition.

[38] Rayner K., Li X., Juhasz B.J., Yan G. (2005). The Effect of Word Predictability on the Eye Movements of Chinese Readers. Psychon. Bull. Rev..

[39] Kennedy A., Pynte J., Murray W.S., Paul S.-A. (2013). Frequency and Predictability Effects in the Dundee Corpus: An Eye Movement Analysis. Q. J. Exp. Psychol..

[40] Miellet S., Sparrow L., Sereno S.C. (2007). Word Frequency and Predictability Effects in Reading French: An Evaluation of the E-Z Reader Model. Psychon. Bull. Rev..

[41] Cui L., Zang C., Xu X., Zhang W., Su Y., Liversedge S.P. (2022). Predictability Effects and Parafoveal Processing of Compound Words in Natural Chinese Reading. Q. J. Exp. Psychol..

[42] Rayner K., Well A.D. (1996). Effects of Contextual Constraint on Eye Movements in Reading: A Further Examination. Psychon. Bull. Rev..

[43] Rayner K., Warren T., Juhasz B.J., Liversedge S.P. (2004). The Effect of Plausibility on Eye Movements in Reading. J. Exp. Psychol. Learn. Mem. Cogn..

[44] Rayner K., Slattery T.J., Drieghe D., Liversedge S.P. (2011). Eye Movements and Word Skipping during Reading: Effects of Word Length and Predictability. J. Exp. Psychol. Hum. Percept. Perform..

[45] White S.J., Rayner K., Liversedge S.P. (2005). The Influence of Parafoveal Word Length and Contextual Constraint on Fixation Durations and Word Skipping in Reading. Psychon. Bull. Rev..

[46] Bélanger N.N., Rayner K. (2013). Frequency and Predictability Effects in Eye Fixations for Skilled and Less-Skilled Deaf Readers. Vis. Cogn..

[47] Siegelman N., Schroeder S., Acartürk C., Ahn H.-D., Alexeeva S., Amenta S., Bertram R., Bonandrini R., Brysbaert M., Chernova D. (2022). Expanding Horizons of Cross-Linguistic Research on Reading: The Multilingual Eye-Movement Corpus (MECO). Behav. Res. Methods.

[48] Mor B., Prior A. (2022). Frequency and Predictability Effects in First and Second Language of Different Script Bilinguals. J. Exp. Psychol. Learn. Mem. Cogn..

[49] Berzak Y., Levy R. (2023). Eye Movement Traces of Linguistic Knowledge in Native and Non-Native Reading. Open Mind.

[50] Kuperman V., Siegelman N., Schroeder S., Acartürk C., Alexeeva S., Amenta S., Bertram R., Bonandrini R., Brysbaert M., Chernova D. (2023). Text Reading in English as a Second Language: Evidence from the Multilingual Eye-Movements Corpus. Stud. Second Lang. Acquis..

[51] Quiñonez-Beltran J.F., Seymour T.M., Robbins R.A.J., Xu Y., Joshi R.M. (2024). What Can Eye Movements Tell Us about Reading in a Second Language: A Scoping Review of the Literature. Educ. Sci..

[52] Martin K.I., Juffs A. (2021). Eye-tracking as a window into assembled phonology in native and non-native reading. J. Second Lang. Stud..

[53] Blythe H.I., Joseph H.S.S.L. (2011). Children’s Eye Movements during Reading. Oxf. Handb. Eye Mov..

[54] Blythe H.I., Häikiö T., Bertam R., Liversedge S.P., Hyönä J. (2011). Reading Disappearing Text: Why Do Children Refixate Words?. Vis. Res..

[55] Barnes A.E., Kim Y.-S. (2016). Low-Skilled Adult Readers Look like Typically Developing Child Readers: A Comparison of Reading Skills and Eye Movement Behavior. Read. Writ. Interdiscip. J..

[56] Kuperman V., Van Dyke J.A. (2011). Effects of Individual Differences in Verbal Skills on Eye-Movement Patterns during Sentence Reading. J. Mem. Lang..

[57] Berzak Y., Nakamura C., Smith A., Weng E., Katz B., Flynn S., Levy R. (2022). CELER: A 365-Participant Corpus of Eye Movements in L1 and L2 English Reading. Open Mind.

[58] Berzak Y., Katz B., Levy R. Assessing Language Proficiency from Eye Movements in Reading. Proceedings of the Proceedings of the 2018 Conference of the North American Chapter of the Association for Computational Linguistics: Human Language Technologies.

[59] Whitford V., Titone D. (2012). Second-Language Experience Modulates First- and Second-Language Word Frequency Effects: Evidence from Eye Movement Measures of Natural Paragraph Reading. Psychon. Bull. Rev..

[60] Whitford V., Titone D. (2016). Eye Movements and the Perceptual Span during First- and Second-Language Sentence Reading in Bilingual Older Adults. Psychol. Aging.

[61] Whitford V., Titone D. (2017). The Effects of Word Frequency and Word Predictability during First- and Second-Language Paragraph Reading in Bilingual Older and Younger Adults. Psychol. Aging.

[62] Fernandez L.B., Allen S.E.M. (2025). Reduced Capacity for Parafoveal Processing (ReCaPP) Leads to Differences in Prediction Between First and Second Language Readers of English. J. Eye Mov. Res..

[63] Xiao X.Z., Jia G.D., Wang A.P. (2023). Semantic Preview Benefit of Tibetan-Chinese Bilinguals during Chinese Reading. Lang. Learn. Dev..

[64] Daniels P.T., Share D.L. (2018). Writing System Variation and Its Consequences for Reading and Dyslexia. Sci. Stud. Read..

[65] Tsai J.-L., McConkie G.W. (2003). Where Do Chinese Readers Send Their Eyes?. The Mind’s Eye: Cognitive and Applied Aspects of Eye Movement Research.

[66] Yen M.-H., Radach R., Tzeng O.J.-L., Hung D.L., Tsai J.-L. (2009). Early Parafoveal Processing in Reading Chinese Sentences. Acta Psychol..

[67] Inhoff A.W., Liu W., Tang H. (1999). Use of prelexical and lexical information during Chinese sentence reading: Evidence from eye movement studies. Reading Chinese Script: A Cognitive Analysis.

[68] Wang C.-A., Tsai J.-L., Inhoff A.W., Tzeng O.J.L. (2009). Acquisition of Linguistic Information to the Left of Fixation during the Reading of Chinese Text. Lang. Cogn. Process..

[69] Wong K.F.E., Chen H.-C. (1999). Orthographic and Phonological Processing in Reading Chinese Text: Evidence from Eye Fixations. Lang. Cogn. Process..

[70] Bai X., Yan G., Liversedge S.P., Zang C., Rayner K. (2008). Reading Spaced and Unspaced Chinese Text: Evidence from Eye Movements. J. Exp. Psychol. Hum. Percept. Perform..

[71] Inhoff A.W., Wu C. (2005). Eye Movements and the Identification of Spatially Ambiguous Words during Chinese Sentence Reading. Mem. Cogn..

[72] Huang H.-W., Lee C.-Y., Tsai J.-L., Lee C.-L., Hung D.L., Tzeng O.J.-L. (2006). Orthographic Neighborhood Effects in Reading Chinese Two-Character Words. NeuroReport.

[73] Sui L., Woumans E., Duyck W., Dirix N. (2025). The Word Frequency Effect in First- and Second-Language Reading by Chinese and Dutch Bilinguals. Bilingualism.

[74] Li X., Pollatsek A. (2020). An Integrated Model of Word Processing and Eye-Movement Control during Chinese Reading. Psychol. Rev..

[75] Chen P.-H., Tsai J.-L. (2015). The Influence of Syntactic Category and Semantic Constraints on Lexical Ambiguity Resolution: An Eye Movement Study of Processing Chinese Homographs. Lang. Linguist..

[76] Liversedge S.P., Drieghe D., Li X., Yan G., Bai X., Hyönä J. (2016). Universality in Eye Movements and Reading: A Trilingual Investigation. Cognition.

[77] Liversedge S.P., Olkoniemi H., Zang C., Li X., Yan G., Bai X., Hyönä J. (2024). Universality in Eye Movements and Reading: A Replication with Increased Power. Cognition.

[78] Kerek E., Niemi P. (2009). Learning to Read in Russian: Effects of Orthographic Complexity. J. Res. Read..

[79] Siegelman N., Schroeder S., Bao Y.B., Acartürk C., Agrawal N., Bolliger L.S., Brasser J., Campos-Rojas C., Drieghe D., Filipović Đurđević D. (2025). Wave 2 of the Multilingual Eye-Movement Corpus (MECO): New Text Reading Data across Languages. Stud. Second Lang. Acquis..

[80] Alexeeva S.V., Slioussar N.A. (2017). Parafoveal Processing in Reading: The Role of Word Length. Tomsk State Univ. J. Philol..

[81] Staroverova V., Lopukhina A., Zdorova N., Ladinskaya N., Vedenina O., Goldina S., Kaprielova A., Bartseva K., Dragoy O. (2023). Phonological and Orthographic Parafoveal Processing during Silent Reading in Russian Children and Adults. J. Exp. Child Psychol..

[82] Mashanlo T.E. (2018). The Effect of L2 Proficiency on the Eye Movement Measures during L2 Reading in Russian-Chinese and Chinese-Russian Late Bilinguals. Tomsk State Univ. J..

[83] Norkina M., Alexeeva S., Chernova D., Harchevnik M. (2024). A sentence corpus for learners of Russian as a foreign language: The impact of universal parameters on lexical access in a non-native language. Russ. Linguist..

[84] Korneev A., Akhutina T., Matveeva E. An eye-tracking study of reading in russian primary school children. Proceedings of the Conference: Cogninitive Science in Moscow.

[85] Averyanova G.N., Belikova L.G., Erofeeva I.N. (1999). Standardized Tests in Russian as a Foreign Language. Third Certification Level. General Proficiency.

[86] Schotter E.R., Dillon B. (2025). A Beginner’s Guide to Eye Tracking for Psycholinguistic Studies of Reading. Behav. Res. Methods.

[87] Bates D., Mächler M., Bolker B., Walker S. (2015). Fitting Linear Mixed-Effects Models Using Lme4. J. Stat. Softw..

[88] Kuznetsova A., Brockhoff P.B., Christensen R.H.B. (2017). lmerTest Package: Tests in Linear Mixed Effects Models. J. Stat. Softw..

[89] Wickham H. (2016). Data Analysis. ggplot2: Elegant Graphics for Data Analysis.

[90] Hohenstein S., Kliegl R. (2014). Semantic Preview Benefit during Reading. J. Exp. Psychol. Learn. Mem. Cogn..

[91] Bosma E., Nota N. (2020). Cognate facilitation in Frisian–Dutch bilingual children’s sentence reading: An eye-tracking study. J. Exp. Child Psychol..

[92] Friesen D.C., Jared D. (2007). Cross-language message- and word-level transfer effects in bilingual text processing. Mem. Cogn..

